# Increased posterior cingulate cortex blood flow in cancer-related fatigue

**DOI:** 10.3389/fneur.2023.1135462

**Published:** 2023-07-27

**Authors:** David Raizen, Rupal Bhavsar, Brendan T. Keenan, Patrick Z. Liu, Timothy P. Kegelman, Hann-Hsiang Chao, Neha Vapiwala, Hengyi Rao

**Affiliations:** ^1^Department of Neurology, Perelman School of Medicine, University of Pennsylvania, Philadelphia, PA, United States; ^2^Division of Sleep Medicine, Department of Medicine, Perelman School of Medicine, University of Pennsylvania, Philadelphia, PA, United States; ^3^Chronobiology and Sleep Institute, University of Pennsylvania, Philadelphia, PA, United States; ^4^Department of Radiation Oncology, ChristianaCare, Newark, DE, United States; ^5^Radiation Oncology Service, Richmond VA Medical Center, Richmond, VA, United States; ^6^Department of Radiation Oncology, Virginia Commonwealth University, Richmond, VA, United States; ^7^Department of Radiation Oncology, Perelman School of Medicine, University of Pennsylvania, Philadelphia, PA, United States; ^8^Shanghai Key Laboratory of Brain-Machine Intelligence for Information Behavior, Center for Magnetic Resonance Imaging Research, Shanghai International Studies University, Shanghai, China

**Keywords:** fatigue, cancer, default mode network, functional MRI, posterior cingulate cortex, sleep, cerebral blood flow, arterial spin labeling

## Abstract

Fatigue is a common symptom associated with cancer treatments. Brain mechanisms underlying cancer-related fatigue (CRF) and its progression following therapy are poorly understood. Previous studies have suggested a role of the default mode network (DMN) in fatigue. In this study we used arterial spin labeling (ASL) perfusion functional magnetic resonance imaging (fMRI) and compared resting cerebral blood flow (CBF) differences in the posterior cingulate cortex (PCC), a core hub of the DMN, between 16 patients treated with radiation therapy (RAT) for prostate (9 males) or breast (7 females) cancer and 18 healthy controls (HC). Resting CBF in patients was also measured immediately after the performance of a fatiguing 20-min psychomotor vigilance task (PVT). Twelve of 16 cancer patients were further followed between 3 and 7 months after completion of the RAT (post-RAT). Patients reported elevated fatigue on RAT in comparison to post-RAT, but no change in sleepiness, suggesting that the underlying neural mechanisms of CRF progression are distinct from those regulating sleep drive progression. Compared to HC, patients showed significantly increased resting CBF in the PCC and the elevated PCC CBF persisted during the follow up visit. Post-PVT, but not pre-PVT, resting CBF changes in the PCC correlated with fatigue changes after therapy in patients with CRF, suggesting that PCC CBF following a fatiguing cognitive task may be a biomarker for CRF recovery.

## Introduction

Fatigue is a common symptom that impairs cancer patients' quality of life. Cancer-related fatigue (CRF) is reported by patients as cognitive, physical, and/or emotional exhaustion that interferes with normal functioning (1). A majority of cancer patients suffer from CRF (2–4), making this a high-priority area of research (5). Previous studies have reported increased fatigue during radiation therapy (RAT) in patients with various types of cancer (6–8).

In considering the neurobiological basis of the symptom of fatigue, one key question is whether there are specific neural circuits altered in the fatigue state or, alternatively, there is global brain dysfunction. A promising approach to answering this question involves the use of functional magnetic resonance imaging (fMRI) methods for non-invasively measuring brain activity and functional connectivity changes associated with fatigue.

Recent neuroimaging studies have suggested the important role of the default mode network (DMN) in fatigue, both in cancer patients [for a review of other brain changes involved in CRF (5)] and other fatiguing illnesses (9–13). The DMN is a resting-state brain network involved in intrinsic, self-referential and stimulus-independent thoughts with higher activity at rest and lower activity during performing goal-directed cognitive tasks (14). In healthy subjects, impaired resting-state DMN activity and connectivity are associated with mental fatigue following fatiguing cognitive task performance (9). Altered DMN activity and connectivity have also been reported in various clinical populations in which fatigue symptoms are pervasive, including breast cancer (10, 11), multiple sclerosis (12), and Parkinson's disease (13).

A core component of the DMN is the posterior cingulate cortex (PCC) (14). Prominent within the DMN, the PCC in the resting state has connectivity changes to the cerebellum and middle temporal gyrus in the context of CRF (11). Furthermore, there is increased connectivity in the PCC associated with Parkinson's disease-related fatigue (15). However, it is unclear whether altered DMN connectivity, particularly within the PCC, corresponds to increased blood flow to the region in the context of CRF.

Another key question is whether the symptom of fatigue and its progression with cancer therapy can be explained by sleepiness and sleepiness progression. While attention is reduced both in the sleep deprived state (16) and in the non-sleep-deprived fatigue state (17), it is unclear to what degree CRF mimics the sleep-deprived state. While cancer patients report both excessive daytime sleepiness and fatigue (18), whether sleepiness can explain CRF and its progression remains unclear.

To assess these questions, we used arterial spin labeling perfusion fMRI to measure cerebral blood flow in the PCC in cancer patients undergoing radiation therapy (RAT) and compared to healthy controls of similar age and sex. A subset of cancer patients were further followed 3–7 months after completion of the RAT. ASL perfusion imaging provides reliable and non-invasive quantification of regional CBF that is tightly coupled to regional neural activity, making it well suited to study fatigue in CRF. We hypothesized that regional CBF in the PCC will be altered in our CRF subjects as compared to healthy controls and changes in PCC CBF will correlate with changes in CRF from RAT to post-RAT follow up. In addition to fMRI analysis, patients were given a sleepiness questionnaire to explore the connection between post-RAT fatigue progression and sleepiness.

## Methods

### Subjects

A total of 16 radiation therapy patients treated for either non-metastatic breast or non-metastatic prostate cancer participated in this study. There were nine males and seven females (mean age was 57.4, with a range of 34–75 years) enrolled in the study. Subjects were recruited from the Perelman Center for Advanced Medicine while receiving daily external beam radiation for cancer treatment. Five subjects began the study while still receiving daily radiation therapy while seven subjects began the study within 2 weeks of completing the radiation therapy and while still fatigued. Four subjects began the study 2 weeks after completing RAT. All 16 subjects are included in the “on RAT” analysis group. Twelve of these patients returned for a follow-up assessment between 3 and 7 months after completion of RAT [mean (± SD) time between RAT and post-RAT follow-up of 18.9 ± 5.2 weeks (range: 12.3 to 30.1 weeks)].

As part of their routine clinical care, patients underwent a weekly fatigue assessment as a component of the Common Terminology Criteria for Adverse Events (CTCAE) version 4 instrument, reporting fatigue scores ranging 0–3 during treatment. Patients who reported an increase in fatigue on therapy in comparison to their fatigue prior to starting RAT were considered for the study. Patients were included if they were undergoing definitive, adjuvant, or salvage prostate cancer radiation, and post-mastectomy or post-lumpectomy breast radiation. Participants were excluded if they had any of the following: (1) History of untreated symptomatic obstructive sleep apnea (OSA, defined as an apnea-hypopnea index ≥ 15); (2) Score on the Multivariable Apnea Prediction (MAP) algorithm >0.7 (19); (3) History of narcolepsy; (4) Night shift work; (5) Distant metastatic disease at presentation; or (6) Active alcohol or drug dependence. They were also excluded if they had a contraindication for undergoing an MRI.

We analyzed data obtained from an additional 18 non-fatigued healthy controls of similar age and sex (11 males and seven females, mean [±SD] age 55.7 ± 14.7, range: 27–73 years) who had completed the same resting fMRI protocol as that done by patients, except that control subjects had only a single resting ASL scan and no PVT or post-PVT scans.

### Questionnaires

The Brief Fatigue Inventory (BFI) (20) was used to measure fatigue and the Epworth sleepiness scale (ESS) (21) was used to measure sleepiness in patients during RAT and post-RAT. The BFI and ESS were completed only by the patients and not by the healthy controls. A BFI≥4 and ESS>10 are clinically-used thresholds for making treatment decisions with respect to fatigue and excessive sleepiness, respectively (22, 23). The study was conducted in accordance with the University of Pennsylvania Institutional Review Board. Written informed consent was obtained from all the participants.

### Psychomotor vigilance task (PVT)

The psychomotor vigilance task (PVT) (24–26) was used as a 20-min sustained attention task during fMRI. The PVT is a reaction time test with random inter-stimulus intervals (ISI) that range from 2 to 10 s. The mean ISI was 6 s, including a 1 s delay after each button press for subjects to read their reaction time. Participants were instructed to focus their attention on a red, rectangular box subtending 2 × 1.3 degrees of visual angle in the middle of a black screen. They were instructed to stop the counter with a button press as soon as they saw a number displayed, while avoiding false starts. The primary purpose of the PVT was to provide an additional measurement of CBF in the PCC after an acute fatiguing task.

### Imaging data acquisition and analyses

Functional Imaging data were acquired on a Siemens Magnetom 3.0 T Prisma scanner (Siemens AG, Erlangen, Germany), using a 64-channel head coil. All fMRI scans were conducted at the Hospital of the University of Pennsylvania. A 2-shot spiral 3D pseudo-continuous arterial spin labeling (ASL) sequence was used for the perfusion scan with the following parameters: TR = 4s, TE = 10 ms, flip angle = 90°, image matrix = 64 × 64, FOV = 240 mm, labeling time = 1.8 s, post labeling delay = 1.7 s. Because each image was acquired in 2-TRs for this sequence, the effective TR was 8 s. A total of 34 slices with 3.75 mm slice thickness were acquired in an interleaved manner from anterior to posterior. High resolution T1-weighted structural images were acquired using a 3D MPRAGE sequence with the following parameters: TR = 2,400 ms, TE = 2.22 ms, flip angle = 8°, 208 slices with slice thickness of 0.80 mm, image matrix = 300 x 320, FOV = 256 mm. The pre-PVT and post-PVT ASL scan each lasted 4.8 min (288 s) with 36 acquisitions, and was performed with the subject resting in the scanner. The PVT scans lasted for 20 min with 150 acquisitions. The total protocol lasted ~40 min, which included the T1 MPRAGE and preparation scans.

Image data analysis was performed using Statistical Parametric Mapping (SPM 12) software (Wellcome Department of Cognitive Neurology, London) based in Matlab R2012b (Mathworks Inc., Natick, MA, USA). The ASL data processing was performed using fMRI Grocer toolbox (https://www.nitrc.org/projects/fmri_grocer/) and in house scripts (27, 28). Pre-processing steps included motion correction, co-registration, normalization and smoothing. Motion correction was done by aligning all the functional images to the mean image of the time series for each run to correct for the effects of head movements. Realigned images were co-registered to individual subject's own T1 structural image. Then, perfusion weighted image series were generated by pair-wise subtraction of the label and control images. The resulting CBF-weighted images were averaged to obtain a mean CBF image for each condition. The mean CBF image was smoothed using a three-dimensional, 8 mm full width at half maximum Gaussian kernel and normalized to a 2 × 2 × 2 mm3 Montreal Neurological Institute (MNI) template. All the warped mean CBF maps were visually checked for optimal sensitivity, good contrast and intensity. One mean resting CBF image was generated for each of the healthy controls. For each of cancer patients with two visits, four mean resting CBF images were generated for pre-PVT and post-PVT scans during RAT (visit 1) and post-RAT (visit 2), respectively. For each cancer patient and visit, two mean resting CBF images were generated for pre-PVT and post-PVT scans during RAT.

To examine whether PCC CBF were altered in CRF patients as compared with healthy adults, region of interest (ROI) analysis was performed using *a priori* PCC ROI defined from the Automated Anatomical Labeling (AAL) atlas (29, 30). Regional CBF values in the PCC were extracted for patients' mean CBF maps and healthy controls using the Marsbar toolbox (https://marsbar-toolbox.github.io/). PCC CBF changes (after adjusting for global CBF difference) were then calculated and correlated with CRF changes from RAT to post-RAT in patients. Due to individual differences in global CBF values, we adjusted for global CBF of individual subjects when calculating PCC CBF changes. Global CBF values were extracted from the mean CBF images for each subject in SPM. Adjusted PCC CBF was calculated by dividing each subject's regional CBF by their global CBF and multiplying by 50 ml/100 g/min. PCC CBF changes were then calculated by the differences between the adjusted CBF values from RAT to post-RAT.

### Statistical analysis

Continuous data are summarized using means, standard deviations (SDs) and ranges, and categorical data using frequencies and percentages. Paired Student's *T*-tests (continuous data) or McNemar's exact tests (categorical data) were utilized to examine the significance of changes in measurements from RAT to post-RAT or pre-PVT to post-PVT, as well as when comparing differences in two change scores within a given participant. To simultaneously examine whether there were significant changes in PCC CBF between pre/post RAT and pre/post PVT, we utilized a repeated measures analysis of variance (ANOVA). Associations between measurements were assessed using Pearson's linear correlation coefficients (unadjusted) or linear regression (in multivariate analyses). Primary hypotheses/questions of interest included: (i) Are there changes in self-reported measurements of fatigue (BFI) and excessive daytime sleepiness (ESS) several months after RAT in patients with CRF? (ii) Are brain mechanisms, as quantified through CBF in the PCC, different between healthy controls and patients with CRF during or after RAT? (ii) Are there changes in pre-PVT or post-PVT PCC CBF several months after RAT in patients with CRF?; and (iv) Do changes after RAT in pre-PVT or post-PVT PCC CBF associate with magnitude of change in fatigue and sleepiness measures after RAT. We applied a Hochberg step-up correction (31, 32) to control overall type I error at 5% in the context of multiple testing under each of these over-arching hypotheses; any p < 0.05 is considered nominally significant. For correlation analyses, coefficients of 0.1, 0.3, and 0.5 were considered small, medium and large effect sizes based on recommendations from Cohen (33).

## Results

**Figure 1:**
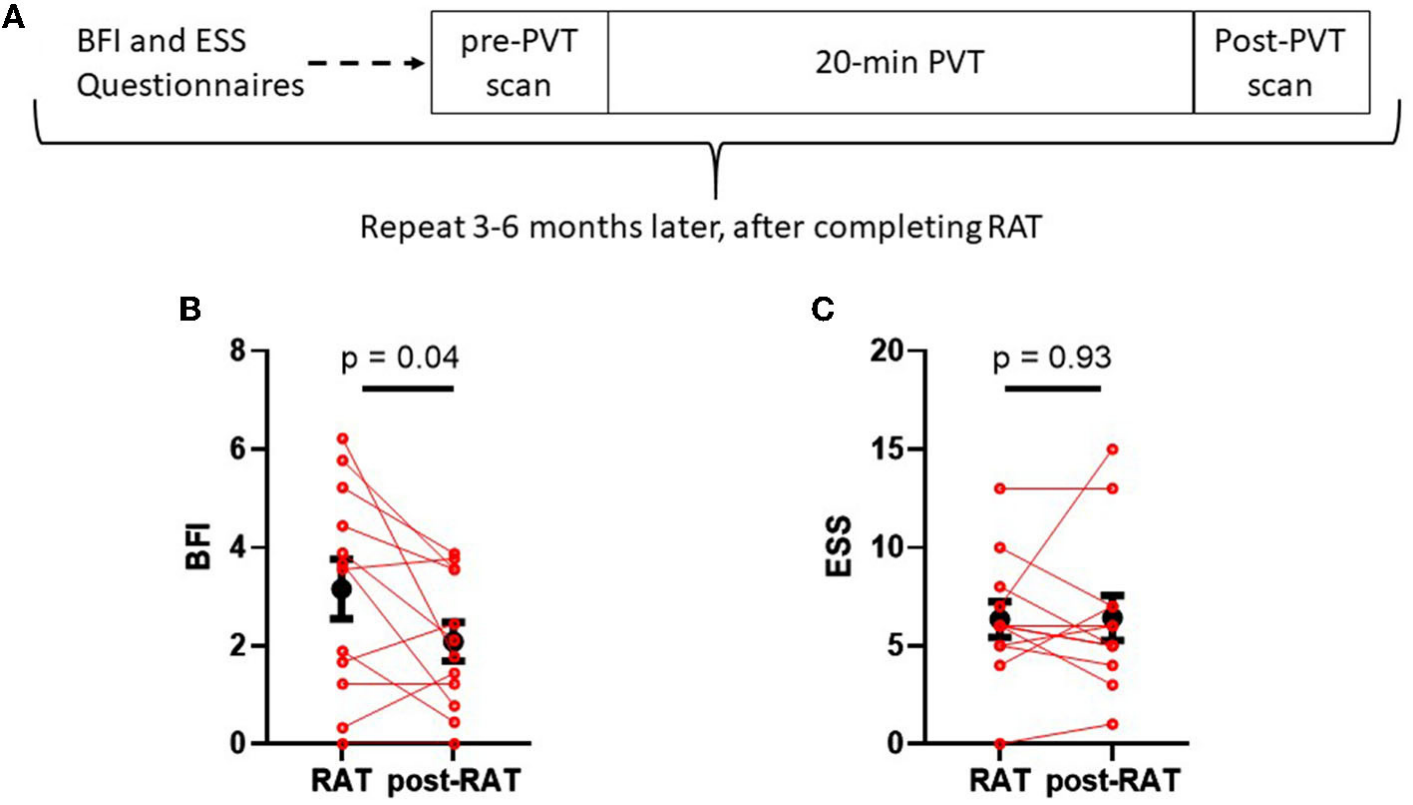
Schematic of study design **(A)** and comparisons between fatigue and sleepiness during RAT to fatigue and sleepiness postRAT **(B, C)**. **(B)** The Brief Fatigue Inventory (BFI) global score was significantly reduced in patients after therapy. **(C)** The Epworth sleepiness scale (ESS) score did not change between RAT and postRAT. Red dots denote values on RAT to those of the same subjects postRAT, connected by a red line. Black dots denote means and error bars denote standard errors of the means. Statistical significance was calculated using a paired Student's T-test.

Figure 1A shows the study design. Participants were recruited from the radiation oncology clinic if they reported increased fatigue from baseline (prior to initiation of radiation therapy) during RAT treatment of cancer (prostate or breast). Subjects completed the brief fatigue inventory and Epworth sleepiness scale questionnaires at the time of enrollment. fMRI studies were performed during the daytime. After undergoing the first resting scan, the subjects completed a 20-min psychomotor vigilance task. A second resting fMRI scan was then performed. Between 12 and 30 weeks after completion of radiation therapy, participants underwent a repeat of the protocol including the questionnaires, PVT, and fMRI.

It is feasible that measurements obtained after RAT or changes from RAT to post-RAT may be influenced by the total time elapsed since RAT treatment. To examine this possibility, we examined the correlation between weeks between measurements with post-RAT values and changes in BFI, ESS, and PCC CBF. As shown in Supplementary Table S1, there were no statistically significant correlations between duration and post-RAT values or changes. Although not statistically significant, there were moderately large correlations between longer duration between measurements and both larger BFI increases (rho = 0.31) and larger ESS decreases (rho = −0.37), suggesting possibly opposite impacts (e.g., increased fatigue but decreased sleepiness) of longer time between RAT and post-RAT measures.

Figure 1B displays Brief Fatigue Inventory measures from each patient during radiation therapy (RAT) and 10–12 weeks after radiation therapy (post-RAT) (22). Mean BFI score during therapy was 3.15 ± 0.61, and after therapy it decreased to 2.08 ± 0.39 (*p* = 0.042, paired Student's *T*-test); this result did not maintain statistical significance after Hochberg correction. Figure 1C displays Epworth Sleepiness Scale during therapy and after therapy. During therapy, mean ESS score was 6.33 ± 0.92 and after therapy it was 6.42 ± 1.14. There was no significant difference in sleepiness when comparing RAT to post-RAT (*p* = 0.93).

The observed changes in the BFI may be meaningful when compared to established clinical thresholds. BFI global scores of ≥4 are indicative of potential need for fatigue related interventions (22). Within our sample during RAT, 6 of 16 patients (37.5%) met this criteria, whereas post-RAT no patients had a BFI≥4 (McNemar's exact test *p* = 0.125). On the other hand, only 2 of 16 patients (12.5%) met the clinically-accepted threshold for excessive daytime sleepiness of ESS>10 (23) during RAT. Similarly, at follow-up 2 of 12 patients (16.7%) were excessively sleepy; 1 patient who was also excessively sleepy during RAT and 1 who developed excessive sleepiness from RAT to post-RAT (McNemar's exact test *p* > 0.99).

**Figure 2:**
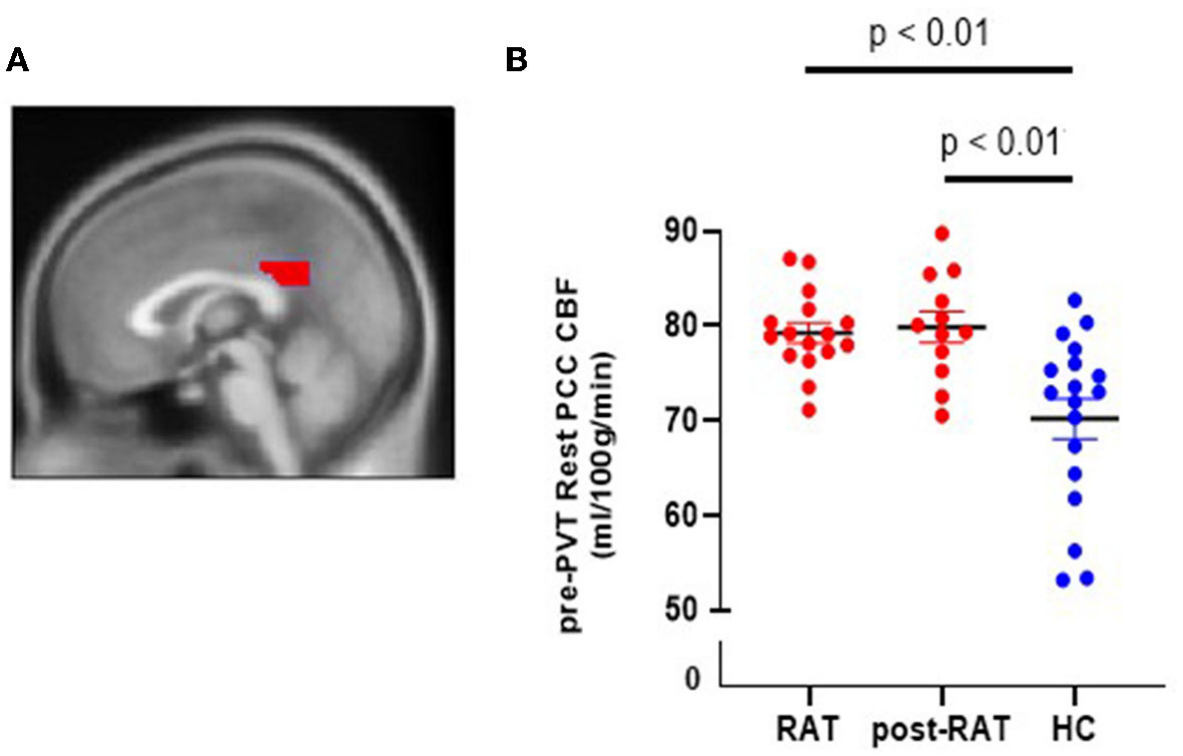
Cerebral blood flow in the posterior cingulate cortex (PCC) in RAT patients and in healthy controls (HC). **(A)** Sagittal view of PCC. **(B)** Pre-PVT resting CBF in the PCC of RAT patients (red) and of healthy controls (blue) during therapy (RAT) and after therapy (postRAT).

Primary neuroimaging studies of fatigue have shown differences in blood flow and connectivity of the default mode network (DMN) (9, 12, 34). A key component of the DMN is the posterior cingulate cortex (PCC) (14). We therefore hypothesized that cerebral blood flow to the PCC will be altered in our CRF subjects. To test this hypothesis, we measured CBF to the PCC (defined using automated anatomical labeling or AAL) in CRF subjects as well as in healthy controls. Figure 2A shows the posterior cingulate cortex (PCC), a region of interest acquired from the AAL toolbox. Figure 2B shows PCC CBF during the 1st rest period prior to performing the PVT (pre-PVT) during therapy (RAT) as well as after therapy (post-RAT) and its comparison to healthy controls. Mean (±SD) PCC CBF in patients was 79.27 ± 4.21 ml/100 g/min during RAT and 79.87 ± 5.58 ml/100 g/min post-RAT, both of which were statistically significantly higher than mean PCC CBF (70.21 ± 9.01 ml/100 g/min) in healthy controls (both p < 0.01). However, there were no differences in the pre-PVT PCC CBF (*p* = 0.86) or post-PVT PCC CBF (*p* = 0.49) during RAT and post-RAT among patients with CRF.

There were also no statistically significant changes in PCC CBF from pre-PVT to post-PVT either during RAT (*p* = 0.47) or post-RAT (*p* = 0.28). In complementary analyses, we evaluated these effects in a single mixed effects model (accounting for the repeated measures within subject) that combined all CBF measurements. Results are consistent with the above analyses, supporting no significant difference pre-PVT vs. post-PVT (*p* = 0.475) or RAT vs. post-RAT (*p* = 0.793) on average, as well as no meaningful interaction (e.g., no difference in the pre-PVT to post-PVT change during RAT or post-RAT and no difference in the change from RAT to post-RAT during pre-PVT or post-PVT; *p* = 0.588).

**Figure 3:**
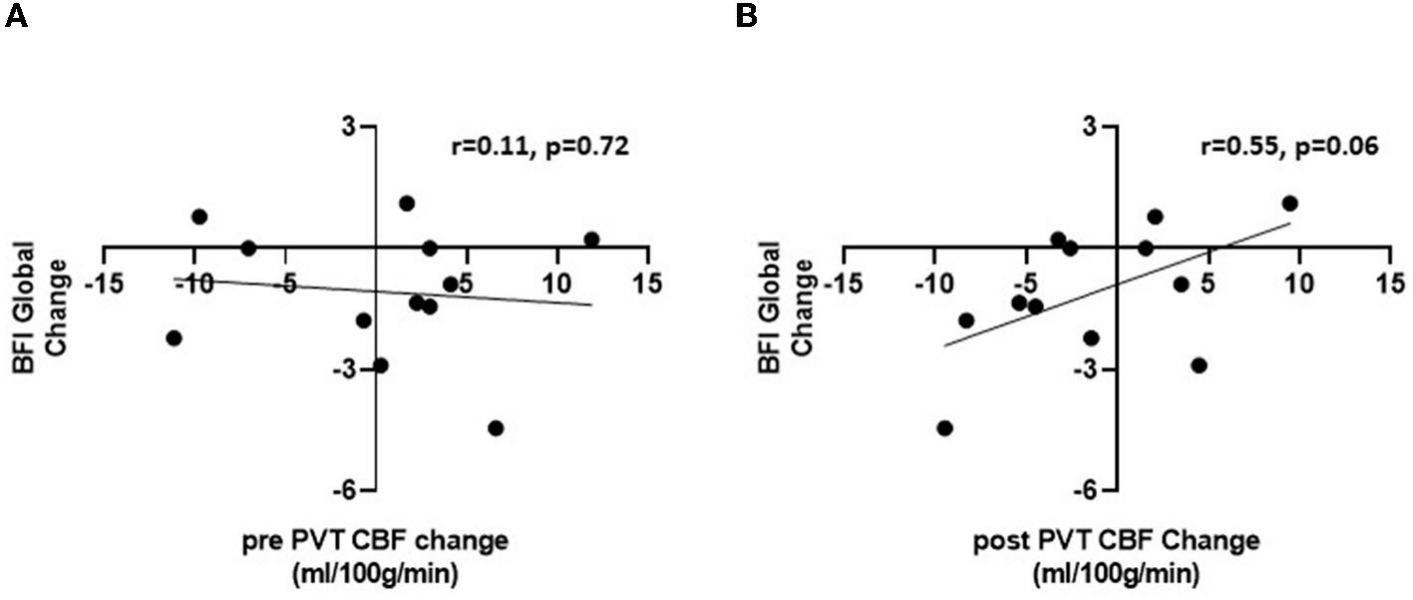
Pearson correlations between change in BFI global fatigue score between visits 1 and 2 and change in PCC CBF during pre-PVT **(A)** and post-PVT resting scans **(B)**.

Although the mean PCC CBF remained elevated at the post-RAT visit, we hypothesized that the change in fatigue severity from RAT to post RAT may correlate with the change in PCC CBF. We first tested this correlation for PCC CBF during the pre-PVT rest period, and found no correlation (rho = −0.11, *p* = 0.72, see Figure 3A). We then reasoned that completing the PVT, which we have shown is cognitively fatiguing (35, 36), may amplify the degree of fatigue and the relationship between fatigue and PCC CBF in individual subjects. After completing the PVT, there was a trend toward significant and large correlation (rho=0.55, *p* = 0.066) between fatigue change and PCC CBF change from RAT to post-RAT (Figure 3B). As the fatigue improved, the cerebral blood flow in the PCC was reduced. This result remained consistent in a multivariate analysis that simultaneously evaluated the association between pre-PVT and post-PVT changes in PCC CBF and change in fatigue from RAT to post-RAT; no association was found for pre-PVT (*p* = 0.917), but a trending positive association with the post-PVT PCC CBF change score (β [95% CI] = 0.162 [-0.029, 0.353]; *p* = 0.087). There were no significant correlations between change in ESS and change in either pre-PVT PCC CBF (rho=0.35, *p* = 0.260) or post-PVT PCC CBF (rho=0.26, *p* = 0.419).

## Discussion

In this study, we reproduced an observation reported frequently in the literature: patients receiving radiation therapy for cancer report elevated levels of fatigue during or shortly after the completion of therapy. Whereas most prior studies assess fatigue at a single time point during RAT, in our study we assessed fatigue twice: once during RAT (or within 2 weeks of RAT completion) and once several months later, when the fatigue has resolved to a variable extent. Utilizing this longitudinal study design (Figure 1A) with repeated measures of questionnaires and CBF within subjects enhances statistical power and can reduce potential confounding.

The complaint of fatigue is often reported by individuals with sleep disorders or healthy individuals after sleep curtailment (37). Here, we tested the hypothesis that sleepiness, as captured by the validated and widely-used Epworth Sleepiness Scale (ESS), is reported by patients receiving RAT before resolving months after completion of RAT. In contrast to the complaint of fatigue, which decreased months after RAT, we found no change in the ESS on RAT when compared to several months post RAT. Therefore, the complaint of elevated fatigue during RAT appears to capture something different from the complaint of sleepiness. The implication of this observation is that the underlying biology explaining fatigue may be different from the biology explaining sleepiness.

The default mode network [DMN, reviewed in Buckner et al. (38)] is composed of several connected brain regions that activate during passive task states as compared to active states (when the subject is performing a specific task). The DMN is believed to activate during internal modes of cognition (38). Inputs from the subsystems of the DMN, such as the medial temporal lobe and the medial frontal lobe subsystems, converge onto nodes of integration including the posterior cingulate cortex (PCC). Therefore, activation of the PCC is central to the activation of the DMN. In support of our hypothesis, we observed increased blood flow to the PCC in fatigued individuals compared to healthy controls.

This elevated CBF to the PCC persisted in the cancer patients, even after their fatigue had reduced. At first glance, this observation may suggest that increased CBF to the PCC is a brain biomarker for cancer and cancer treatment in general, rather than for cancer fatigue specifically. However, we observed a strong positive correlation (rho = 0.55, *p* = 0.066) (33) between the change in fatigue scores and the change in CBF from RAT to post-RAT, but only by measuring resting CBF after completion of a 20-min PVT. If this observation is reproduced in future studies, it would suggest that the performance of a fatiguing mental task (such as the PVT) may amplify any borderline effects on PCC activation as a marker of fatigue.

The increased PCC CBF is consistent with other studies that reported the effects of fatigue related neuronal activity in breast cancer and Multiple Sclerosis (MS) patients. DMN hyper-connectivity has been shown in a group of fatigued breast cancer subjects who completed cancer treatments (11). DMN connectivity may drive fatigue in the absence of depressive symptoms, and higher DMN connectivity was associated with higher fatigue symptom burden in patients with multiple sclerosis (12, 39). Taken together, these findings support a key role of DMN in fatigue. However, we do not yet know whether the observed PCC CBF changes are part of the mechanism of fatigue or, instead, reflect a compensatory mechanism to maintain cognitive function when fatigued.

Our study had limitations. Most importantly, the number of subjects we studied was small and therefore offered us limited statistical power; validation of any positive associations seen in our data in larger and independent samples is needed to ensure robustness. Given our limited sample, we were only able to test a directed neuroimaging hypothesis (change in PCC CBF). Future studies with larger sample sizes may allow us to proceed with a more agnostic discovery approach as to the brain regions whose activity changes in the fatigued state. In addition, larger samples may in the future allow us to explore the influence of other characteristics, such as tumor type and stage, age, and medication use, on regional CBF.

Second, we assessed sleepiness using a subjective tool, the Epworth Sleepiness Scale (ESS). While the ESS has been well validated, it does not provide objective data. Performing a multiple sleep latency test in the future may give a more objective assessment of subject sleepiness.

Third, cancer patients had resting ASL scans both before and after the fatiguing PVT, while healthy controls had only one ASL resting scan without PVT. Although a previous ASL study in healthy young adults showed no differences in resting PCC CBF before and after the 20 min PVT performance (32), future studies are needed to examine the effects of the fatiguing PVT on resting PCC CBF in healthy controls with different age ranges and compared with cancer patients.

Since fatigue is mainly a brain complaint and the site of irradiation (prostate in our male participants and breast in our female participants) in our CRF patients is outside the brain, there must be a communication between genotoxic injury to peripheral tissue and the observed brain function change in the PCC. Such communication could occur via neural signaling, cytokine signaling, a change in metabolites, or some combination of the three. Future studies are necessary to further understand the potential roles of these pathways in connecting peripheral injury with the elevated PCC CBF.

To our knowledge, this is the first report of elevated resting PCC CBF in cancer patients during RAT, which persisted 3–7 months after completion of the RAT. Moreover, post-PVT resting CBF changes in the PCC correlated with fatigue changes during follow up, suggesting that PCC CBF measured by ASL following a fatiguing cognitive task may be a potential biomarker for CRF remission.

## Data availability statement

The raw data supporting the conclusions of this article will be made available by the authors, without undue reservation.

## Ethics statement

The studies involving human participants were reviewed and approved by University of Pennsylvania Institutional Review Board. The patients/participants provided their written informed consent to participate in this study.

## Author contributions

Study was designed by DR, HR, and NV. Subjects were recruited by H-HC, TK, and NV. Imaging was performed by RB. Data was analyzed by DR, RB, HR, and BK. The manuscript was drafted and edited by DR, RB, PL, HR, and BK. All authors contributed to the article and approved the submitted version.
