# Supplementary material for: Increased posterior cingulate cortex blood flow in cancer-related fatigue
Source: Front Neurol. 2023 Jul 27;14:1135462. doi: 10.3389/fneur.2023.1135462 (PMC10413554; doi:10.3389/fneur.2023.1135462)
Supplement: Supplementary file 1 [file Table_1.docx]

Supplementary Table S1: Correlation of weeks between MRI and BFI Global, ESS, Pre- and Post-PVT PCC CBF

| **Measure** | | **N** | **Pearson Correlation** | |
| --- | --- | --- | --- | --- |
|  |  |  | **rho** | **p** |
| **BFI Global** | Post-RAT | 12 | -0.09 | 0.773 |
|  | Change | 12 | 0.31 | 0.336 |
| **ESS** | Post-RAT | 12 | -0.28 | 0.373 |
|  | Change | 12 | -0.37 | 0.242 |
| **Pre-PVT PCC CBF** | Post-RAT | 12 | 0.16 | 0.621 |
|  | Change | 12 | -0.09 | 0.792 |
| **Post-PVT PCC CBF** | Post-RAT | 12 | 0.13 | 0.684 |
|  | Change | 12 | 0.22 | 0.495 |
